# Supplementary material for: Sex and Age Differences in a Progressive Synucleinopathy Mouse Model
Source: Biomolecules. 2023 Jun 11;13(6):977. doi: 10.3390/biom13060977 (PMC10296285; doi:10.3390/biom13060977)
Supplement: Supplementary file 1 [file biomolecules-13-00977-s001.zip › biomolecules-2370625-supplementary.pdf]

Supplementary Material

Table S1. Primary and secondary antibodies

**Primary antibodies**	**Host**	**Company**	**Catalog #**	**Dilution**
Human α-Synuclein	Rabbit	Abcam	Ab138501	1/500
DAT	Rat	Millipore	MAB369	1/250
GFAP	Rabbit	Dako	Z0334	1/500
Iba1	Goat	Novus Biological	NB100-1028	1/1000
TH	Sheep	Abcam	Ab113	1/200
**Secondary antibodies**	**Host**	**Company**	**Catalog #**	**Dilution**
Anti-Goat Alexafluor 633	Donkey	Life Technologies	A21082	1/1000
Anti-Rabbit Alexafluor 488	Donkey	Life Technologies	A21206	1/1000
Anti-Rat Texas Red	Donkey	LifeSpan Biosciences	LS-C61063-2000	1/1000
Anti-Sheep Cy3	Donkey	Millipore	AP184C	1/800

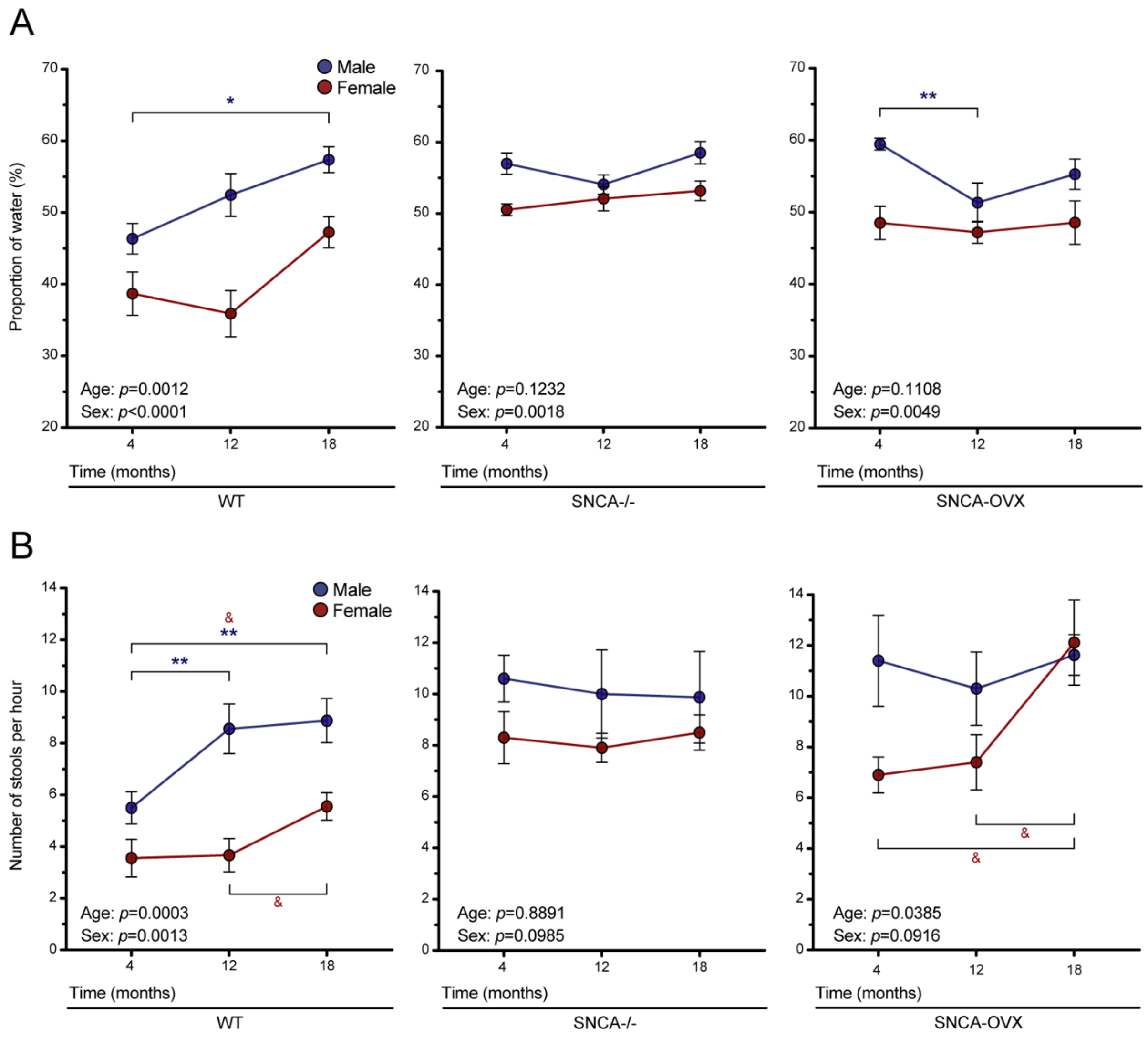

Figure S1. Sex differences in the efficiency of stool excretion. A. Analysis of the proportion of water in feces of mice reveals that male excretes a higher level of fluid that female mice for all genotypes. **B**. The count of stools per hour shows that wildtype male and SNCA-OVX female mice have a faster increase of fecal excretion than the opposite sex. Each group included 8-10 mice. Tukey's post-hoc tests: (Male) *$p < 0.05$, **$p < 0.01$; (Female) $^{\&}p < 0.05$. **Abbreviations:** WT, wildtype.

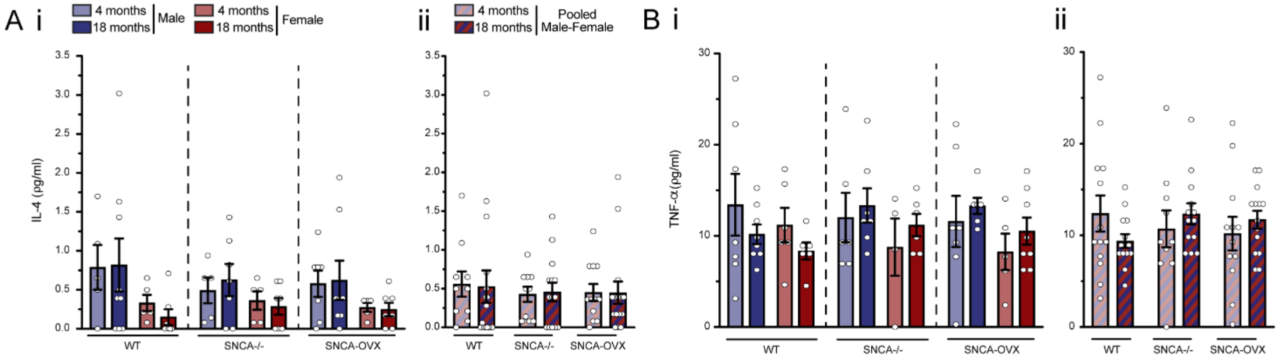

Figure S2. Aging and aSyn have no effect on IL-4 and TNF-α levels. A-B. Plasma analysis of IL-4 (**A**) and TNF-α (**B**) reveals non-significant change with aging or between animal models. Although IL-4 appears higher in male than female, no sexual differences were found. Each group included 7-10 mice. **Abbreviations:** IL, interleukin; TNF-α, tumor necrosis factor alpha; WT, wildtype.
